# Hypoglycaemia avoidance behaviour and exercise levels in active youth with type 1 diabetes

**DOI:** 10.1002/edm2.153

**Published:** 2020-05-30

**Authors:** Alissa J. Roberts, Joyce P. Yi‐Frazier, Kristen Carlin, Craig E. Taplin

**Affiliations:** ^1^ Seattle Children’s Hospital Division of Endocrinology and Diabetes Seattle WA USA; ^2^ University of Washington Seattle WA USA; ^3^ Seattle Children’s Research Institute Seattle WA USA; ^4^ Present address: Perth Children’s Hospital Nedlands WA 6009 Australia

**Keywords:** exercise, hypoglycaemia, type 1 diabetes mellitus

## Abstract

**Aims:**

The primary goal of this exploratory study was to examine the association between fear of hypoglycaemia (FOH), hypoglycaemia avoidance behaviours and exercise in active youth with type 1 diabetes (T1D).

**Methods:**

30 youth with T1D who participate in some physical activity (PA), age 15.0 ± 2.4 years, on insulin pump therapy completed the ‘Type 1 Diabetes Report of Exercise Practices Survey (T1D‐REPS)’ and parent and child hypoglycaemia fear surveys (HFS). Twenty‐eight participants completed the 3‐day PA recall survey. Clinical data and pump downloads were obtained at the time of the survey collection.

**Results:**

Higher child HFS behaviour and total scores were associated with higher PA levels (*P* = .003, *P* = .027), and higher parent HFS behaviour score was associated with higher youth PA levels (*P* = .031), after adjusting for age, sex, duration of diabetes and BMI. Higher child HFS behaviour score was associated with a higher exercise hypoglycaemia avoidance score on T1D‐REPS (*r* = .38, *P* = .043). Higher child HFS worry and total scores were associated with higher HbA1c (*r* = .48, *P* = .008; *r *= .46, *P* = .012).

**Conclusions:**

This study demonstrated that, in a generally active cohort of youth with T1D, increased hypoglycaemia avoidance behaviour was associated with higher PA levels. Higher overall FOH scores were associated with PA level, driven by higher behaviour subscale scores, while worry subscales were not correlated with PA level. Those with more FOH intervene more to specifically avoid exercise‐associated hypoglycaemia and appear to have worse overall glycaemic control. Thus, improved education is required to improve glycaemic control around exercise while maintaining avoidance of hypoglycaemia.

## Graphical abstract

In youth with type 1 diabetes, fear of hypoglycaemia may be a barrier to physical activity, but little is known about whether FOH impacts the amount of exercise, or whether FOH impacts optimal glycaemic management around exercise. Higher physical activity level was associated with increased reported exercise‐specific hypoglycaemia avoidance behaviour and with overall fear of hypoglycaemia in youth with type 1 diabetes who perform some activity. Active youth report employing strategies to reduce hypoglycaemia risk with exercise, but this may be at the expense of excessive hyperglycaemia. Improved education around strategies to safely exercise without permissive hyperglycaemia is needed.

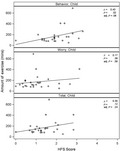

**What is already known?**

- In youth with type 1 diabetes, fear of hypoglycaemia may be a barrier to physical activity, but little is known about whether FOH impacts the amount of exercise, or whether FOH impacts optimal glycaemic management around exercise.

**What this study has found?**

- Higher physical activity level was associated with increased reported exercise‐specific hypoglycaemia avoidance behaviour and with overall fear of hypoglycaemia in youth with type 1 diabetes who perform some activity.

**What are the clinical implications of this study?**

- Active youth report employing strategies to reduce hypoglycaemia risk with exercise, but this may be at the expense of excessive hyperglycaemia.
- Improved education around strategies to safely exercise without permissive hyperglycaemia is needed.

## 1 INTRODUCTION

Fear of hypoglycaemia (FOH) is common in parents and youth with type 1 diabetes (T1D).^1^ Some level of fear may be protective to prevent hypoglycaemia, a dangerous complication of diabetes management.^2^ However, fear may also contribute to acute and chronic hyperglycaemia, and thus risks of diabetes complications; for example an association between parental FOH and poor glycaemic control in youth with T1D has been reported.^3^, ^4^ Child FOH may also be associated with poor glycaemic control^1^; however, some studies show no adverse correlation with HbA1c.^5^

Fear of hypoglycaemia overall is cited as a prominent barrier to physical activity (PA) in adults and youth with T1D.^6^, ^7^, ^8^ Exercise may be a major cause of severe hypoglycaemia in youth with T1D,^2^ though in several countries recently reported rates of severe hypoglycaemia are lower than previously seen, perhaps in part due to modern diabetes management such as insulin pump therapy and continuous glucose monitoring.^9^, ^10^ A qualitative study in adults with T1D suggested that low levels of knowledge and lack of confidence around managing diabetes around exercise was the most notable diabetes‐specific barrier to exercise, along with nondiabetes‐related barriers, and fear of hypoglycaemia was not a raised as a primary concern.^11^

It remains unclear whether FOH impacts exercise management behaviours in an adaptive (promoting more monitoring and appropriate adjustments around exercise) or maladaptive (eg over consumption of carbohydrates and/or purposeful hyperglycaemia) manner. The goal of this exploratory study was, therefore, to explore the relationship between (a) FOH and exercise management strategies in active youth with T1D and (b) FOH and physical activity level. We hypothesized that higher FOH would be associated more insulin adjustments and other key behaviours around exercise specifically to avoid hypoglycaemia, as well as with lower PA levels.

## 2 METHODS

### 2.1 Participants and procedures

Youth were eligible for participation if they were ages 10‐19 years, had T1D for ≥2 years, on an insulin pump as their primary treatment regimen for at least three consecutive months, involved in any PA at least once per week at the time of the study, had a BMI < 95th percentile, were not a ward of the state and could read and write in English. Scheduled clinic patients were screened via chart review for eligibility. Consecutive potentially eligible youth were initially approached in‐person during their clinic visit to determine if their PA status qualified them to participate (any activity done at least once a week would qualify, including gym class, organized sports participation, walking and yoga class).

Informed consent and assent were reviewed and signed by the parent and/or participant. Participants were then given a study packet. For participants’ ages 10‐13 years, parents could assist in filling out the questionnaires, except for the child hypoglycaemia fear survey (CHFS) which was completed by the participant. They were given a $10 gift card upon completion of the surveys. All aspects of the study followed a protocol approved by the Seattle Children's Research Institute Institutional Review Board.

### 2.2 Measures

The parent hypoglycaemia fear survey (PHFS) and child hypoglycaemia fear survey (CHFS), both of which have demonstrated reliability and construct validity,^1^, ^12^ were completed by one parent and the child. Permission was obtained from the survey authors to use this for research purposes. The PHFS and CHFS consist of two components—the worry subscale (15 items) and the behaviour subscale (10 items for the CHFS, 11 for PHFS). Each item is scored from 0 to 4, 0 being consistent with least fear of hypoglycaemia and 4 with most. An average per item score is generated and results in behaviour and worry subscale mean scores, and a total mean score. The 3‐day physical activity recall tool (3DPAR), previously validated^13^ and used in other studies of exercise in youth with type 1 diabetes,^14^ was administered. This tool asks the participant to recall activity on 3 days in the previous week‐ one weekend day, and two weekdays, by assigning an activity as well as effort level for each 30 minutes interval of the day. Activities and effort levels stated all correspond with a standardized Metabolic Equivalent of Task (MET) as per the tool's standard scoring key.

The previously published tool ‘Type 1 Diabetes Report of Exercise Practices Survey’ (T1D‐REPS)^15^ to assess behaviours around exercise was administered. Using nine key questions with rated responses from 1 to 3 (1 being least hypoglycaemia avoidance, 3 being most avoidance) focusing on target exercise blood glucose, basal insulin adjustments, prandial insulin adjustments, bedtime carbohydrate and nocturnal insulin adjustments (Appendix S1), a Hypoglycemia Avoidance Score was generated. Thus, the possible range of scores was 9‐27:9 indicating least hypoglycaemia avoidance behaviour around exercise, and 27 indicating the highest level of exercise hypoglycaemia avoidance.

Clinical information including HbA1c, BMI, age, sex, date of diagnosis, date of pump initiation and insurance type was obtained via chart review, with the reference point being the clinic visit at study enrolment. HbA1c was measured by DCA Vantage. A paired glucometer and pump download were collected at the clinic visit, with a minimum of 14 days of data required for analysis. The blood glucose average, number of glucose checks, average total daily insulin and number of hypoglycaemia events (<70 mg/dL, 3.9 mmol/L) were recorded.

### 2.3 Data analysis

Descriptive statistics, including means, standard deviations, frequencies and percentages, were calculated. All continuous variables were assessed for normality using histograms and QQ‐plots. Independent samples *t* tests were used to assess differences in mean HFS scores if youth made insulin to carbohydrate ratio adjustments, suspended insulin pumps during exercise, reduced overnight basal rate, adjusted insulin to account for a bedtime snack, and if youth had a continuous glucose monitor. HFS scores were also compared between target glucose levels (120‐180 and 180‐250) using independent samples *t* tests. Pearson or Spearman correlation coefficients were used to assess the association of Hypoglycemia Avoidance Score with CHFS scores, HbA1c, PA levels, rate of hypoglycaemia, frequency of glucose checks and duration of diabetes.

Multivariable linear regression models were used to assess the impact of fear of hypoglycaemia on total exercise. Residual plots were used to assess model assumptions and the assumption of heteroscedasticity was violated. Thus, a log‐transformation was applied to the dependent variable (total exercise). Estimates from the model were then exponentiated for interpretability and can be interpreted as the ratio of the geometric mean of exercise time when comparing two groups of youth that differ by one unit in the HFS scores. The False Discovery Rate (FDR) was used to account for multiple comparisons. Both raw and FDR‐adjusted *P*‐values are reported. Significance testing was done at the α = .05 level. SAS 9.4 (SAS Institute Inc) was used for all other analyses.

## 3 RESULTS

### 3.1 Demographic characteristics

**Figure 1:**
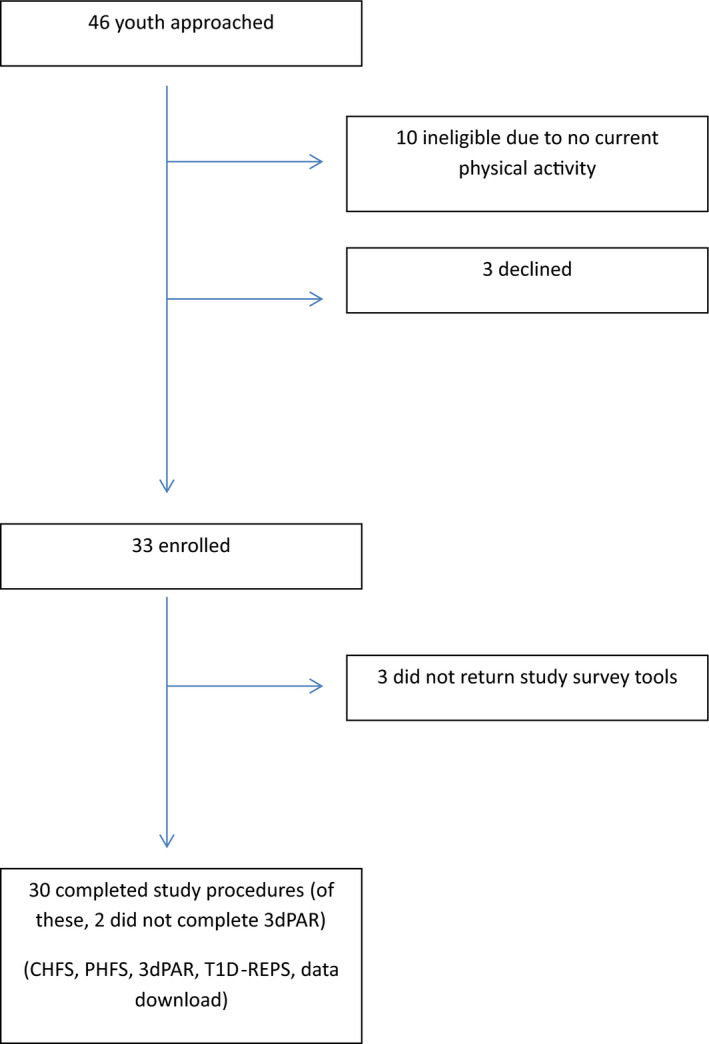
Study recruitment

Forty‐six youth were approached. Ten of these youth were ineligible based on lack of any reported PA (no data could be obtained about exercise management), and a further three declined to participate. Thirty‐three were thus enrolled; 30 completed the required questionnaires and were included in the analysis (N = 30) (Figure 1). Of note, 2 of these 30 did not complete the 3DPAR, but were included in analyses that did not incorporate 3DPAR data. Participant characteristics are presented in Table 1. Ninety‐three percent of participants had an average of ≥60 minutes of PA (moderate/vigorous) per day on 3DPAR, meeting national recommendations for PA in youth. Table 2 presents mean HFS scores in our study population.

**Table 1:** Participant characteristics

	Mean (SD)	Range
Age (y)	15.0 (2.4)	10.4‐19.1
BMI (*z*‐score)	0.5 (0.6)	−0.8 to 1.7
HbA1C (%)	8.7 (1.0)	7.2‐11.4
HbA1C (mmol/mol)	72 (11)	55‐101
Duration of diabetes (y)	9.9 (3.4)	2.7‐17.1
Duration of pump use (y)	6.7 (2.6)	1.4‐13.0
Glucose checks/d	5.3 (2.7)	1.4‐11.4
Blood glucose readings < 70 mg/dL (%)	4.1 (3.0)	0‐13.2
Number of episodes of severe hypoglycaemia in past 12 mo	0^a^ (25th%tile = 0, 75th%tile = 1)	0‐5
Average daily physical activity (min)	165 (138) 125^a^ (25th%tile = 90, 75th%tile = 175)	0‐690
Average daily metabolic equivalent (MET)	71.9 (13.0)	48.0‐101.0
≥60 min Average daily physical activity	26/28 (93%)	
Sex	13 (43%) female	
CGM use	9 (30%) often, 3 (10%) rarely	

a Median reported.

**Table 2:** Hypoglycemia fear survey scores

	Mean (SD)	Range
CHFS‐B	2.00 (0.67)	0‐3.30
CHFS‐W	1.06 (0.72)	0‐3.13
CHFS‐T	1.42 (0.63)	0‐2.84
PHFS‐B	2.22 (0.58)	1.09‐3.00
PHFS‐W	1.35 (0.56)	0.53‐2.87
PHFS‐T	1.72 (0.45)	1.04‐2.77

Abbreviations: CHFS‐B, Child hypoglycaemia fear survey‐behaviour; CHFS‐W, Child hypoglycaemia fear survey‐worry; CHFS‐T, Child hypoglycaemia fear survey‐total; PHFS‐B, Parent hypoglycaemia fear survey‐behaviour; PHFS‐W, Parent hypoglycaemia fear survey‐worry; PHFS‐T, Parent hypoglycaemia fear survey‐total.

### 3.2 Child FOH and exercise management

CHFS behaviour score was associated with Exercise Hypoglycemia Avoidance Score (*r* = .38, *P* = .043). No single/specific behaviours were associated with FOH: insulin adjustments for pre or postexercise meal, basal rate suspension or reduction during exercise, overnight basal rate reduction and bedtime snack insulin adjustments. No differences were seen in FOH or hypoglycaemia avoidance score by continuous glucose monitor use.

### 3.3 Child FOH and physical activity level

**Figure 2:**
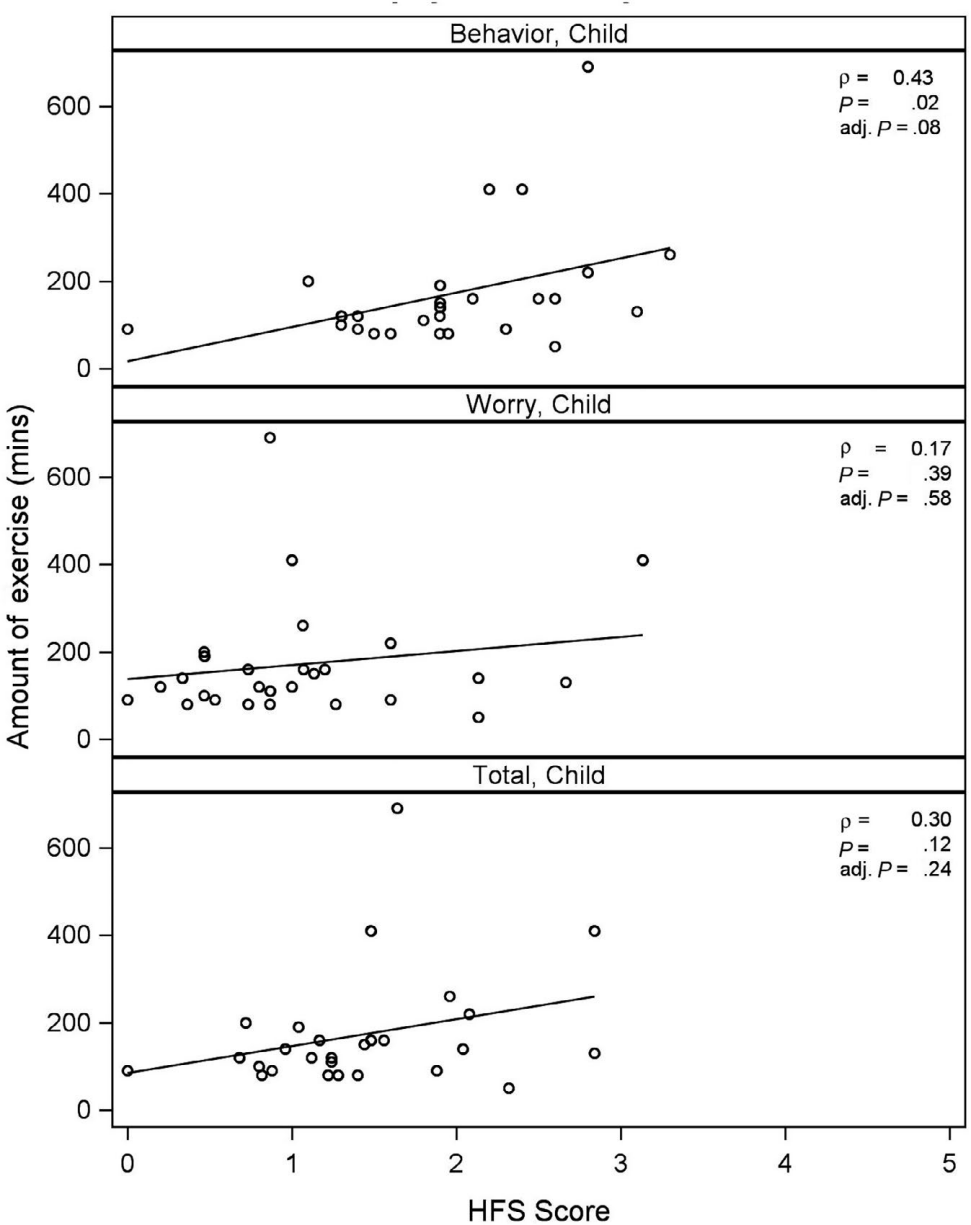
Unadjusted association between nonlogarithm transformed total physical activity and CHFS behaviour scales using Spearman correlation

Higher child HFS behaviour and total scores were associated with higher PA levels, after adjusting for patient age, sex, duration of diabetes and BMI (Table 3 and Figure 2). A single unit increase in the CHFS behaviour score was associated with an increase of 50% in the time spent exercising when compared to one CHFS behaviour scale unit lower (*P* = .003). Expressed with respect to SD, for each 1 SD increase of CHFS behaviour score, exercise increased by 34%. A similar pattern was seen with CHFS total scores; for each 1 SD increase of CHFS total score, exercise increased by 26% (*P* = .027). Child HFS worry scores were not significantly associated with PA level. Higher exercise Hypoglycemia Avoidance Scores generated from T1D‐REPS were associated with higher PA levels (*r* = .60, *P* < .001).

**Table 3:** Linear regression results showing the association of HFS scores with amount of exercise

Type of scale	Unadjusted			Adjusted^a^			
	Estimate	95% CI	*P*‐value	Estimate	95% CI	Raw *P*‐value	FDR adj. *P*‐value
Behaviour, Child	1.41	1.03‐1.94	.034	1.50	1.16‐1.94	.003	.021
Worry, Child	1.14	0.83‐1.57	.397	1.25	0.94‐1.65	.123	.184
Total, Child	1.29	0.91‐1.84	.149	1.41	1.04‐1.91	.027	.063
Behaviour, Parent	1.25	0.83‐1.90	.276	1.51	1.04‐2.20	.031	.063
Worry, Parent	1.08	0.70‐1.68	.722	1.11	0.73‐1.68	.606	.606
Total, Parent	1.23	0.73‐2.08	.418	1.39	0.86‐2.25	.164	.197

Note

Estimates are presented as exponentiated β and are interpreted as the ratio of the geometric mean of exercise time when comparing two groups of patients that differ by one unit in the HFS scores.

a Adjusted for age, sex, years since diagnosis and BMI.

### 3.4 Child FOH and glycemic control

**Figure 3:**
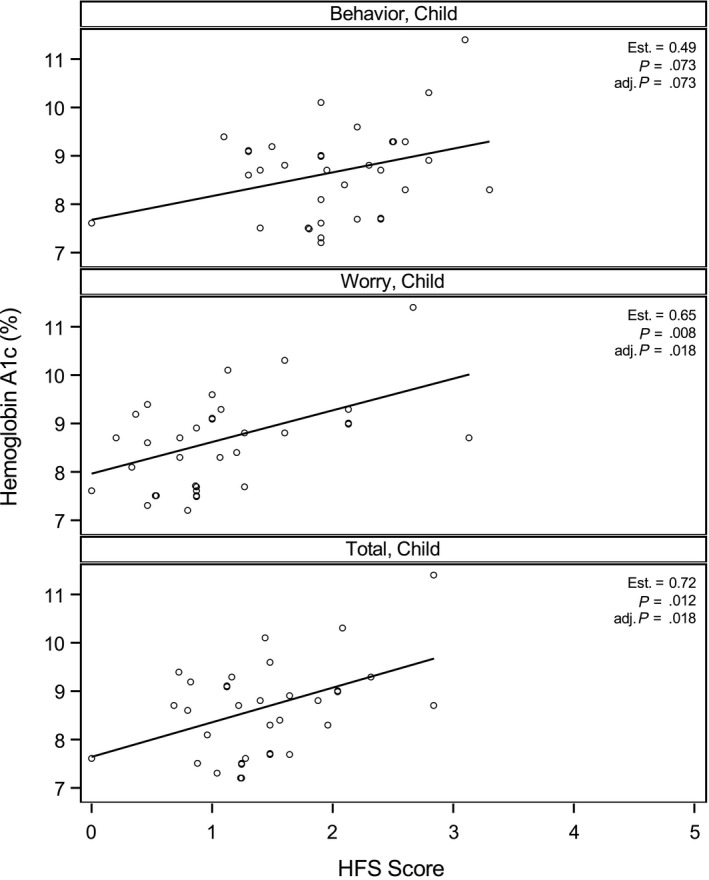
Unadjusted association between haemoglobin A1c (%) and CHFS behaviour scales using Pearson correlation

Higher CHFS worry and total scores were moderately correlated with HbA1c (Figure 3) (worry: *r* = .48, *P* = .008; total: *r* = .46, *P* = .012) indicating that more youth FOH is associated with higher HbA1c. CHFS behaviour scores were not correlated with HbA1c. No association was found between FOH and rate of hypoglycaemia, frequency of glucose checks or duration of diabetes (Table 4).

**Table 4:** Correlation between CHFS scores and clinical variables

	Behaviour CHFS score			Worry CHFS score			Total CHFS score		
	*r*	Raw *P*‐value	FDR‐adj. *P*‐value	*r*	Raw *P*‐value	FDR‐adj. *P*‐value	*r*	Raw *P*‐value	FDR‐adj. *P*‐value
Hypoglycemia avoidance score	.38	.043	.105	.22	.256	.407	.27	.153	.276
HbA1c	.34	.073	.164	.48	.008	.061	.46	.012	.065
Years since diagnosis	.01	.949	.986	.02	.938	.986	−.00	.990	.986
Rate of hypoglycaemia	.03	.884	.986	−.12	.554	.680	−.03	.879	.986
Glucose readings per day	−.12	.531	.680	−.16	.411	.584	−.13	.525	.680
Behaviour, Parent	.39	.040	.105	.25	.205	.346	.31	.107	.222
Worry, Parent	.42	.025	.076	.42	.025	.076	.48	.009	.061
Total, Parent	.50	.007	.061	.43	.024	.076	.50	.007	.061

### 3.5 Parental FOH

Higher parent HFS behaviour scores were associated with higher youth PA level, when controlling for patient age, sex, duration of diabetes and BMI (Table 3). Similarly to the CHFS score, a for each 1 SD increase of PHFS behaviour score, exercise increased by 30% (*P* = .031). Parent HFS worry and total scores were not associated with youth PA level. Parent HFS scores did not correlate with specific youth exercise behaviours or glycaemic control. Parent and Child HFS scores were moderately correlated (Table 4) (behaviour: *r* = .39, *P* = .040; worry: *r* = .42, *P* = .025; total: *r* = .50, *P* = .007).

## 4 DISCUSSION

In this study, higher hypoglycaemia fear scores, driven by the behaviour subscale and exercise‐specific hypoglycaemia avoidance behaviour were seen in youth with T1D who were most physically active. Reports to date examining this relationship have primarily focused on self‐reported barriers to PA but not assessed associated PA levels by a validated measure.^7^, ^16^ One explanation for these findings is that those who are most physically active are most aware of the risks of hypoglycaemia, have more experience in avoiding it, and thus engage in more hypoglycaemia avoidant behaviours. This may reflect improved education around exercise safety and would explain the observed association with higher FOH behaviour subscale (and thus total FOH) scores. Other potential explanations include that FOH may be less significant as a subjective barrier to exercise than once was the case, but we cannot make this conclusion as this would require a larger study and the inclusion of sedentary youth.

In the era of modern diabetes management, especially in those managing T1D with pump therapy and/or continuous glucose monitors, barriers to an active lifestyle such as hypoglycaemia risk may be balanced for many by the now well‐documented medical and psychosocial benefits of exercise. With widespread use of social media, the higher profile of key organizations (eg Diabetes Sports Project) and multiple evidence‐based consensus statements published recently, many forums and modalities now exist for encouraging physical activity for youth with T1D. Indeed, several professional athlete role models have been open regarding their experiences; the sum total of this may be shifting attitudes toward a more inclusive and active exercise goal for all youth with T1D.

Of note, our study population met daily PA requirements (based on the last week and 3DPAR) at rates (93%) comparable to, but higher than the SEARCH for Diabetes in Youth study which found 82% of youth with T1D were active for at least 60 minutes a day using the same assessment tool.^14^ Mean scores on the child and parent HFS in our study (Table 2) correspond with mean HFS scores previously reported in the literature for youth with T1D and their parents,^1^ supporting the potential generalizability of this study.

Concerning findings in this study include that higher FOH was associated with higher HbA1c. Some awareness of hypoglycaemia risk around exercise may be beneficial and productive in maintaining euglycaemia and preventing severe hypoglycaemia. However, we found that greater fear of hypoglycaemia was associated with poorer overall glucose control, but we cannot state that glucose levels were higher on the day of exercise, as the study design and limitations prevent this conclusion. That higher fear correlates with higher PA and HbA1c, though, suggests a possible link between these constructs and may explain the finding that some, but not all, studies of exercise as an intervention in T1D have not reported improvements in HbA1c. This may also be related to a lack of patient knowledge or confidence in how to effectively avoid hypoglycaemia around exercise without detrimental hyperglycaemia. Indeed, inadequate provider‐driven education in clinic around exercise has recently been reported by people with type 1 diabetes.^17^ We propose that while recognizing a degree of FOH is important for clinicians, addressing the now considerable evidence base with patents in clinic with regard to exercise and glycaemic management is key. We recognize this is a challenge in the busy clinical setting, and efficient in‐clinic tools are needed to improve the quality of exercise education in youth with T1D.^18^

Given that youth in this study were on insulin pump therapy, many pump‐specific tools exist to allow for safe exercise when starting in the target glucose range (eg pre‐emptive basal insulin rate modification, basal suspension, automated insulin adjustment when using hybrid‐closed loop therapy), as well as extra carbohydrate intake and continuous glucose monitoring. Many youth, however, may not know how or when to employ these tools optimally, but rather initiate exercise in the hyperglycaemic range to allow for an exercise‐related fall in glucose, suggesting efforts to improve education and understanding of exercise physiology are needed in youth with T1D. This is consistent with our previous finding that many youth do not take advantage of appropriate insulin adjustments after exercise.^15^ Indeed, authors MacMillan et al have found that discussion of PA and methods to participate in exercise safely are lacking in diabetes clinic.^17^ Stakeholders spoke of limited PA support provided in the current care model and recognized several opportunities for intervention to improve education and comfort around exercise for youth with T1D (and their parents). There are also unique challenges for exercise in youth; it is often less structured, more spontaneous and unpredictable and thus more challenging to manage. We speculate, also, that youth prioritize participation (and thus hypoglycaemia avoidance as the primary glycaemic goal) over euglycaemia or peak performance during exercise; which would arguably be a developmentally appropriate PA goal.

Important limitations to this study include the small sample size, that we only studied adolescents on insulin pumps, that we do not have accelerometer data available to confirm 3DPAR data, and that a minimum PA level was required for eligibility due to the study goal to assess exercise management. The 3DPAR data are subjective, though is a validated tool used in other studies of youth with T1D to assess PA levels. Although by design to answer questions about how FOH impacts exercise behaviour in exercising youth, by excluding overtly sedentary youth this study may not capture those at greatest risk for FOH influencing their behaviours around exercise. Furthermore, given we only included youth on insulin pumps who participate in physical activity, we likely have selected a motivated cohort and results may not be generalizable. Also, we can draw no conclusions about the interaction between parental fear of hypoglycaemia and physical activity in children < 10 years of age with T1D. We also note that 3 individuals in the study engaged in high amounts of moderate physical activity related to long periods of work‐related tasks at moderate exertion. These were all, however, legitimate reports of physical activity, and thus all were included in the analysis. Larger studies, across multiple clinical populations and accounting for a more sedentary cohort should now be conducted to assess these relationships further.

## 5 CONCLUSION

In youth with T1D on insulin pump therapy, more hypoglycaemia avoidant behaviour around exercise correlates with PA level. FOH was associated with increased rates of permissive hyperglycaemia around exercise and overall poorer glycaemic control. This appears to support increased understanding of strategies to prevent dangerous glycaemic excursions around exercise, but improved patient and provider understanding of exercise physiology in T1D is likely required if exercise is to be performed without adverse effects on glycaemic control.

## CONFLICT OF INTEREST

The authors have no relevant conflicts of interest to disclose. Preliminary data from this study were presented at the ADA (American Diabetes Association) in 2017.

## AUTHOR CONTRIBUTIONS

AJR conceptualized and designed the study, coordinated and supervised data collection, and drafted the initial manuscript. JYF contributed to data acquisition and analysis plan and interpretation, conceptualized and designed the study. KC contributed to data acquisition and analysis plan and interpretation. CT conceptualized and designed the study and supervised all aspects of the study as the senior investigator. All authors reviewed, revised and approved the final manuscript. All authors approved the final manuscript as submitted and agree to be accountable for all aspects of the work.

## Supporting information

Appendix S1

## Data Availability

Data available on request due to privacy/ethical restrictions.
