# Supplementary material for: Hypoglycaemia avoidance behaviour and exercise levels in active youth with type 1 diabetes
Source: Endocrinol Diabetes Metab. 2020 May 30;3(3):e00153. doi: 10.1002/edm2.153 (PMC7375118; doi:10.1002/edm2.153)
Supplement: Supplementary file 1 — Appendix S1 [file EDM2-3-e00153-s001.docx]

Appendix 1: Questions used in generating the “Hypoglycemia Avoidance Score”

1. Most commonly, which one of the following ranges do you aim for your blood sugar to be in at the start of exercise? Please mark only one.
   1. 60-120
   2. 120-180
   3. 180-250
   4. Greater than 250
   5. I don’t know
2. If you eat a meal or snack within 1 hour prior to exercising, do you use your usual insulin to carbohydrate ratio?
   1. Yes
   2. No, I give myself less insulin
   3. I don’t know
3. For this snack or meal (from question 9), which one of the following choices best describes what you eat?
   1. Mostly carbohydrates
   2. Mostly protein
   3. A mix of both carbohydrates and protein
   4. Whatever I am in the mood for

1. On average, how often do you check your blood sugar during exercise?
   1. Not at all
   2. About once an hour
   3. More often than once an hour
   4. Less often than once an hour
   5. Only if I feel low or high
   6. Other (write in):
2. After exercise, do you adjust your insulin to carbohydrate ratio for the first meal you eat?
   1. No
   2. Yes, I give myself less insulin
   3. Yes, I give myself more insulin
   4. It depends on my blood sugar
   5. Other (write in):
   6. I don’t know
3. Do you adjust your basal insulin rate at night if you exercised that day? (you might call this using a “temporary basal rate”)
   1. Always
   2. Often
   3. Rarely
   4. Never
   5. It depends on the timing of my exercise
   6. No, because I am active every day so my overnight basal stays the same
4. What is your blood glucose target as you go to bed?
   1. 60-120
   2. 120-180
   3. 180-250
   4. Greater than 250
   5. Other:
   6. I don’t usually check my blood glucose before going to sleep

Grouped questions: generate one (1-3) score from these three with skip logic:

1. Do you suspend your insulin pump’s basal rate around the time of exercise?
   1. No (skip to question 15)
   2. Yes
   3. Sometimes
2. If you do not suspend your basal rate completely prior to exercise, do you adjust your basal insulin rate BEFORE starting to exercise?
   1. No
   2. Yes
   3. Sometimes
3. I don’t know If you do not keep your pump completely suspended during exercise, do you adjust your basal insulin rate DURING exercise?
   - - 1. No
       2. Yes
       3. Sometimes
       4. I don’t know

Grouped questions: generate one (1-3) score from these two with skip logic:

1. When do you have a bedtime snack on days that you have exercised?
   1. Always
   2. Most of the time
   3. Rarely
   4. Never
   5. Only if I am running low
2. If you do have a bedtime snack on days that you exercise, do you cover this with your normal insulin to carbohydrate ratio?
   1. Yes, with my usual insulin to carbohydrate ratio
   2. No, I cover with less insulin or no insulin at all
